# Dental Trauma on whole Body Trauma CT—An underreported finding

**DOI:** 10.1007/s00068-021-01633-z

**Published:** 2021-03-15

**Authors:** Hans-Jonas Meyer, Dominik Schramm, Andreas Gunter Bach, Alexander Eckert, Alexey Surov

**Affiliations:** 1 Department of Diagnostic and Interventional Radiology, University of Leipzig, Leipzig, Germany; 2 Diagnostic and Interventional Radiology, Martin-Luther-University Halle-Wittenberg, Halle (Saale), Germany; 3 Department of Oral and Maxillofacial Surgery, Martin-Luther-University Halle-Wittenberg, Halle (Saale), Germany; 4 Department of Radiology and Nuclear Medicine, Otto-von-Guericke University Magdeburg, Magdeburg, Germany

**Keywords:** Dental trauma, CT, Whole body CT

## Abstract

**Background:**

The prevalence of dental injuries (DI) in polytrauma patients is unknown. The purpose of our study was to identify the frequency of dental injuries on whole body CTs acquired in a trauma setting and to estimate how often they are correctly reported by the radiologist.

**Methods:**

In the time period between 2006 and 2018 the radiological database of one university hospital was screened for whole-body trauma CTs. A total of 994 CTs were identified and re-evaluated.

**Results:**

Dental injuries were identified in 127 patients (12.8% of patients). There were 27 women (21.3%) and 100 men (78.7%) with a mean age of 51.0 ± 18.9 years (range 10–96 years). Regarding localization, most findings involved the molars (*n* = 107, 37.4%), followed by the incisors (*n* = 81, 28.3%), premolars (*n* = 59, 20.6%) and canines (*n* = 39, 13.7%). Most common findings were as follows: luxations (*n* = 49, 45.8%), followed by crown fractures (*n* = 46, 43%), root fractures (*n* = 10, 9.3%), extrusions (*n* = 1, 0.9%), and intrusions (*n* = 1, 0.9%). Only 15 findings (11.8% of all patients with dental injuries) were described in the original radiological reports.

**Conclusion:**

DI had a high occurrence in polytrauma patients. A high frequency of underreported dental trauma findings was identified. Radiologists reporting whole-body trauma CT should be aware of possible dental trauma to report the findings adequately.

## Introduction

Dental injury (DI) is after soft tissue injury one of the most common facial trauma occurrences. It can be found especially often in children and teenagers, most often involving the anterior region [1–3]. DI comprises crown and root fracture, luxation, intrusion, or avulsion [1]. In trauma patients with maxillofacial fractures, the incidence of DI is 13.1% [1]. In most cases, DI can be diagnosed based on clinical signs. Panoramic radiograph and cone-beam computed tomography (CT) are well-established imaging modalities to visualize DI [4, 5]. Panoramic radiograph is the most used technique [4]. Cone-beam CT can be used to better visualize dental structures. These techniques can detect dental root fractures more reliably than multidetector CT, but they are not always easily available in the early assessment of the polytrauma patient [5–8].

Whole-body CT is a widely used imaging modality to identify injuries in multi-trauma patients [9, 10]. The radiologist is challenged to view around 2000 pictures within a short time period and to correctly report pathological findings. At first, possibly lethal injuries need the full attention, such as intracranial hemorrhage, pneumothorax, vessel injuries, and bone fractures and afterwards the CT should be reassessed for other potential clinically relevant findings including dental findings [9]. Therefore, radiologists tend to overlook relevant findings of the teeth resulting in an overall underreporting of dental pathologies to the clinician [7, 8].

Previously, various studies reported non-traumatic clinically relevant incidental findings in whole body CTs [11–15]. These comprise cardiovascular findings, incidental malignant tumors, or inflammation foci [11–15]. However, no study systematically investigated dental injuries on whole-body trauma CT despite its potential common occurrence. Moreover, no study analyzed the frequency of sufficiently reported dental injuries.

Therefore, the aim of the present study was to identify the frequency of dental injuries on whole body CTs acquired in a trauma setting and to estimate how often they are correctly reported by the radiologist.

## Materials and methods

This retrospective study was approved by the institutional ethics board (Martin-Luther University Halle-Wittenberg) and informed consent was waived.

In the time period between 2006 and 2018 the radiological database of one university hospital was screened for whole-body trauma CTs. All whole-body trauma CTs were analyzed within this time period. Isolated maxillofacial trauma patients were not considered in the present analysis.

The primary objective of this study was to estimate the frequency of dental trauma in a whole-body CT scan. As a next step, it was evaluated in the original radiology reports, whether these findings were reported or not by the radiologist.

We used a classification proposed by the World Health Organization comprising the following traumatic events: crown fracture, root fracture, avulsion, extrusion, intrusion, and luxation [3].

### Computed tomography

Computed tomography (Somatom Sensation 64; Siemens, Erlangen, Germany and Toshiba Aquilion 64, Toshiba Medical Systems GmbH, Neuss, Germany) was performed in all patients. In all cases, 60–140 mL of iodinated intravenous contrast medium was given at a rate of 1.5–3.5 mL/s by a power injector (Medtron GmbH, Germany), with a scan delay of 30–90 s after the onset of injection. Typical imaging parameters were 120 kVp, 150–300 mAs, and a slice thickness of 0.8 mm for the neck region with a pitch of 0.6.

### Image analysis

All images were analyzed in digital format on a PACS workstation (Centricity PACS, GE Medical Systems, Milwaukee, Wisconsin, USA). Every CT scan was again re-analyzed by one radiologist with 7 years of general experience to identify possible dental trauma findings blinded to the clinical and radiology reports. The images were evaluated using 3-dimensional reconstructions. In unclear cases, a consensus was made with a consultant radiologist with 17 years of experience.

### Statistical analysis

Statistical analysis was performed using GraphPad Prism 5 (GraphPad Software, La Jolla, CA). Collected data were evaluated by means of descriptive statistics. Continuous variables were expressed as mean ± standard deviation and categorical variables as percentages. Fisher’s exact test was used to test between groups.

## Results

**Fig. 1:**
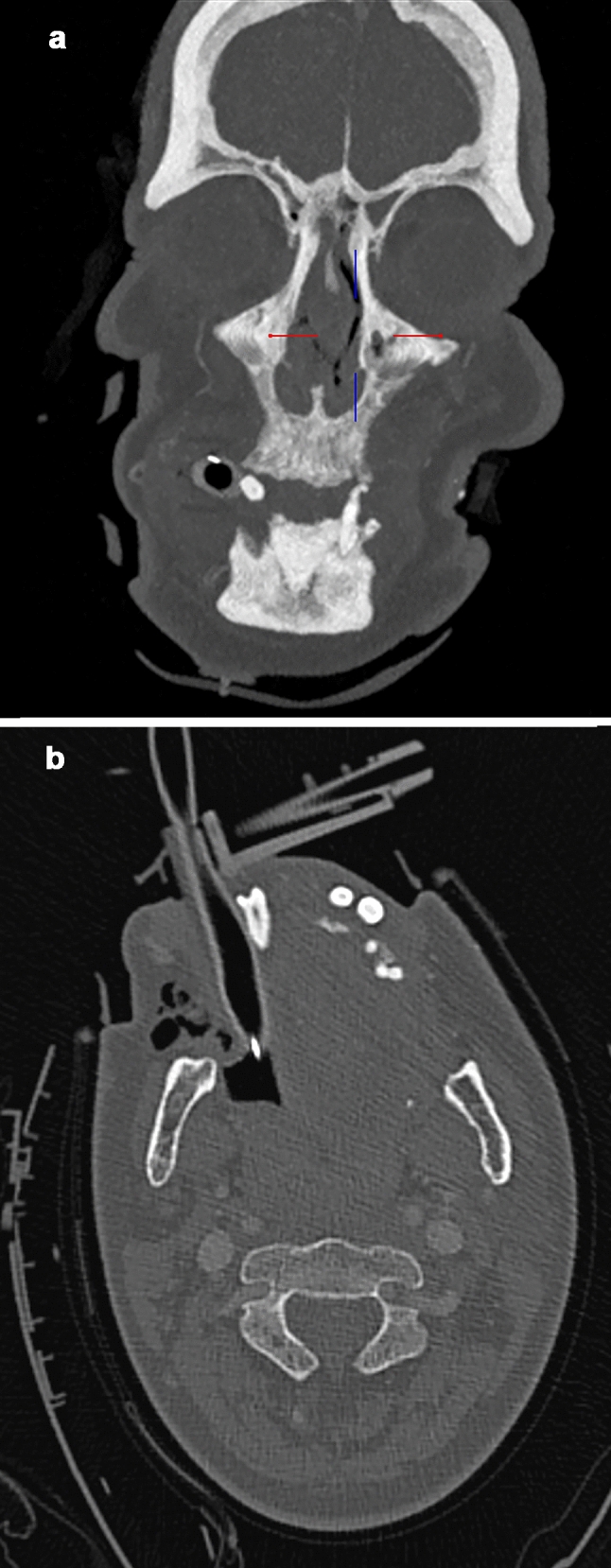
** a** Maximum intensity projection in the coronal plane of a 64-years old male patient. Avulsion of the right lower incisor with an associated non-displaced fracture of the mandible. The tooth is directly adjacent to the tube. **b** Axial plane. The finding was correctly reported by the radiologist

**Fig. 2:**
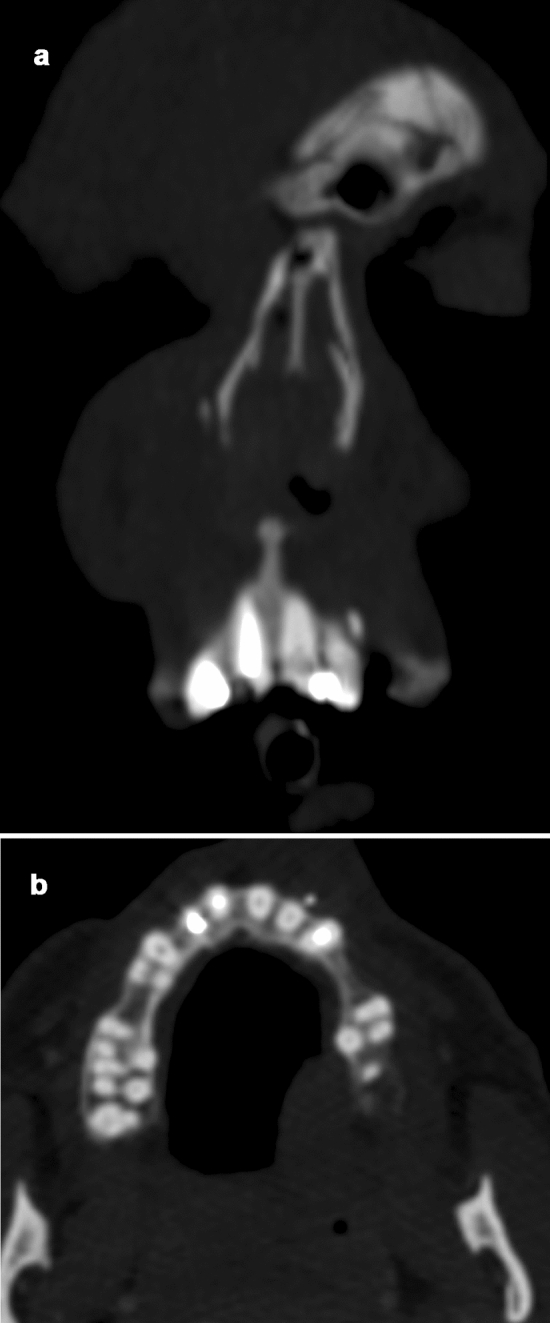
**a** Maximum intensity projection in the coronal plane of a 21-years old male patient. Root fracture of the left upper incisor. The patient suffered from a complex fracture of the skull, yet no direct adjacent fracture to the tooth. This dental finding was not reported by the radiologist. **b** The tooth fragment can also be appreciated in the axial plane

A total of 994 patients/CTs were identified and re-evaluated. Overall, dental trauma injuries occurred in 127 patients (12.8% of all trauma patients) (Figs. 1, 2).

There were 27 women (21.3%) and 100 men (78.7%) with a mean age of 51.0 ± 18.9 years (range 10–96 years). One tooth was injured in 32 cases (41.6%), 2 to 5 teeth were injured in 31 cases (40.1%), and over five teeth were injured in 14 patients (18.2%). Overall, 286 injuries were identified. Furthermore, 41 patients (32.3%) were intubated, and 86 patients (67.7%) were not. In 7 patients (5.5%), an associated bone fracture of the facial region was identified. Regarding localization, most findings involved the molars (*n* = 107, 37.4%), followed by incisors (*n* = 81, 28.3%), premolars (*n* = 59, 20.6%) and canine (*n* = 39, 13.7%) (Table 1).

**Table 1:** Overview about the dental trauma findings identified in all patients

Type of finding	Incisor	Canine	Premolar	Molarn	Total
Crown fracture	28	10	22	46	106
Root fracture	8	7	6	10	31
Avulsion	0	0	0	0	0
Extrusion	1	1	1	1	4
IntrusionIntrusion	0	0	0	1	1
Luxation	44	21	30	49	144
Total	81	39	59	107	

Most common findings were dental luxation (*n* = 49, 45.8%), followed by crown fractures (*n* = 46, 43.0%), root fractures (*n* = 10, 9.3%), extrusions (*n* = 1, 0.9%), and intrusions (*n* = 1, 0.9%). There were no displaced tooth fragments identified within the aerodigestive tract.

Only 15 findings (11.8% of all patients with dental injuries) were identified in the original radiology report (Table 2).

**Table 2:** Dental trauma findings, which were correctly reported by the radiologist

Type of finding	Incisor	Canine	Premolar	Molarn	Total
Crown fracture	3	2	2	4	11
Root fracture	1	5	3	2	11
Avulsion	0	0	0	0	0
Extrusion	0	1	1	1	3
Intrusion	0	0	0	0	0
Luxation	6	9	4	4	23
Total	10	17	10	11	

In the group with correctly reported findings, six patients suffered from an associated facial bone fracture (40%), whereas in the non-reported group only one patient suffered from a fracture (0.9%), which is a significantly different ratio (*p* < 0.001).

## Discussion

The present study identified a high frequency of DI. Overall, DI occurred in 12.8% of all polytrauma cases. Furthermore, only 11.8% of these were sufficiently described in the original radiology reports.

The prevalence of DI in a clinical trauma setting is still unknown. In fact, clinical dental investigations showed that the prevalence of dental trauma ranged significantly, namely from 6 to 59% [16]. Furthermore, in a meta-analysis, a prevalence of dental trauma was up to 5% of all trauma findings [17]. However, no study analyzed the frequency of dental injuries on whole-body CT in trauma patients.

It is not unusual that whole-body CT can detect numerous findings of the body with a high accuracy, comprising non-trauma-related incidental findings and possible hazardous trauma findings [10–14]. However, some dental findings might not be detectable by multidetector CT due to only subtle fracture lines [8]. This is a reason why cone-beam CT has a slight superior accuracy compared to multidetector CT and may detect more trauma findings [18].

Another reason for possible misdiagnosis of DI on whole-body CT can be image artifacts, especially scatter artifacts caused by dental amalgam. These artifacts are a common problem and can obscure the anatomy and potential pathological findings of the oral cavity [19]. Therefore, the frequency of dental injuries might be even higher than reported in this study.

Notably, the radiologist is faced to evaluate numerous images in a small timeframe to make correct diagnoses. In the trauma setting, possible life-threatening conditions must be diagnosed immediately before other imaging findings, including dental-related findings, can be addressed. Then, roughly 40% of patients undergoing a whole-body CT, show additionally at least one incidental finding [11, 12, 20].

This might also be a reason that a lot of findings are not sufficiently reported by the radiologist. For chest CTs, it was acknowledged that only 55% of easily detectable cardiac findings were reported within the radiologist report [21]. Similar results were reported for cardiovascular findings on whole-body CT [14].

In a recent retrospective study, Bulbul et al. reported that dental findings are frequent findings on CT performed to evaluate paranasal sinus [7]. In fact, 51% of patients had a pathological finding, most commonly carious lesions in 27% of cases [7]. In another study examining different head CT scans, it has been shown that dental diseases were significantly underreported with only 11% of sufficient mentions in the radiology report [22]. We identified that dental findings were significantly more reported when DI was associated with a bone fracture. Presumably, the radiologist is more concerned for bone fracture assessment and the fracture leads the radiologist to the dental trauma finding. In short, one key finding of the present study is that dental trauma findings are severely under-reported by radiologists in acute clinical situations.

Unlike dental trauma involving only teeth, which is managed by a dentist in an outpatient clinic, dental trauma associated with polytrauma is managed in a hospital setting [1]. This might also be a reason why the radiologists at a tertiary hospital tends to overlook findings of the teeth. Another reason might be that dental findings tend to be very subtle, easily to be overlooked [8]. Most of the reported findings were severe trauma findings associated with adjacent bone fractures. These findings might be easily detectable and, thus, were reported by the radiologists. Moreover, there might be not enough clinical information regarding trauma mechanism and possible damage of the teeth. Of note, many dental trauma findings can be diagnosed by a clinical examination, except of root fractures, which can only be diagnosed by imaging.

It should be considered that dental trauma may be caused by the initial trauma or may be iatrogenic especially after endotracheal intubation [23, 24]. It was identified that existing dental anomalies increases the risk for dental injury by endotracheal intubation in a 12-fold manner [24]. So far, in a study investigating 3423 emergency endotracheal intubations, only 6 dental injuries (0.2% of all patients) were identified. This finding indicates that the dental injuries detected in the present study were most commonly caused by the trauma itself [24].

On the other hand, dental injuries might complicate the acute treatment itself, especially the endotracheal intubation [25]. Moreover, they could acutely compromise the airway and may lead to aspiration, albeit no systematical data exists investigating such complications. So, a recent case report highlighted the importance of imaging modalities to correctly identify these aspirated tooth fragments [26]. In our patient sample no aspirated tooth fragments were identified, which nevertheless diminished the importance of these findings.

Interestingly, our results are in agreement with a recent epidemiological study, which identified a comparable prevalence (13%) of dental traumas [27]. However, the present results might be different in comparison to those based on clinical examination in patients with isolated dental injuries [28, 29].

There are several important factors for the etiology of dental injuries. Overjet was significantly associated with dental injuries in every dentition and age groups [30]. Moreover, orthodontic treatment is associated with dental injuries in children [31]. In adults, there is a moderate evidence that alcohol use is associated with DI [32]. Previous occurrence of dental injuries is also a risk factor for another one [33].

In summary, the correct diagnosis of dental trauma can be important for the patient and for possible treatment planning. Although not every dental trauma is treated, the cost of dental trauma is high and often time consuming [17].

Our study emphasizes that radiologists need to pay more attention to findings of the teeth in trauma patients due to its high frequency.

There are some limitations of the present study to address. First, it is a retrospective study with possible inherent bias. However, the CTs were evaluated without clinical information and blinded to the radiological and clinical reports to reduce possible bias. Second, the reporting rate is specific for one university hospital. There might be institutions, in which dental trauma findings are reported more frequently. Thirdly, there might be a bias of preexisting dental injuries, which were included in the present analysis as the exact age of dental trauma findings cannot be determined with the CT. Fourthly, the real frequency of dental trauma findings might be even higher due to missed findings on CT.

## Conclusion

DI had a high occurrence in polytrauma patients. A high frequency of underreported dental trauma findings was identified. The radiologists should be aware of possible dental trauma on whole-body trauma CT to sufficiently report these findings.

## Author contributions conceptualization

AS and HJM; methodology: HJM and DS; formal analysis and investigation: HJM, DS, AB, AE; writing—original draft preparation: HJM writing—review and editing: AS; supervision: AS.
